# Spaceflight associated neuro-ocular syndrome (SANS) and the neuro-ophthalmologic effects of microgravity: a review and an update

**DOI:** 10.1038/s41526-020-0097-9

**Published:** 2020-02-07

**Authors:** Andrew G. Lee, Thomas H. Mader, C. Robert Gibson, William Tarver, Pejman Rabiei, Roy F. Riascos, Laura A. Galdamez, Tyson Brunstetter

**Affiliations:** 1 Department of Ophthalmology, Houston Methodist Hospital, Houston, TX USA; 2 Baylor College of Medicine and The Baylor Center for Space Medicine, Houston, TX USA; 3 Departments of Ophthalmology, Neurology, and Neurosurgery, Weill Cornell Medical College, New York, NY USA; 4 Department of Ophthalmology, The University of Texas Medical Branch, Galveston, TX USA; 5 Department of Ophthalmology, The University of Iowa Hospitals and Clinics, Iowa City, IA USA; 6 UT MD Anderson Cancer Center, Houston, TX USA; 7 Colonel (R) US Army, Moab, UT USA; 8 Coastal Eye Associates, Webster, TX USA; 9 Space Flight Associated Neuro-ocular Syndrome (SANS) Clinical Lead, Clinical Services, NASA Johnson Space Center, Houston, TX USA; 10 Department of Diagnostic and Interventional Imaging, University of Texas Health Science Center at Houston, Houston, TX USA; 11 U.S. Navy Detailed to NASA Johnson Space Center, Houston, TX USA; 12 Department of Emergency Medicine, Baylor College of Medicine, Houston, TX USA

**Keywords:** Eye manifestations, Medical research, Physical examination

## Abstract

Prolonged microgravity exposure during long-duration spaceflight (LDSF) produces unusual physiologic and pathologic neuro-ophthalmic findings in astronauts. These microgravity associated findings collectively define the “Spaceflight Associated Neuro-ocular Syndrome” (SANS). We compare and contrast prior published work on SANS by the National Aeronautics and Space Administration’s (NASA) Space Medicine Operations Division with retrospective and prospective studies from other research groups. In this manuscript, we update and review the clinical manifestations of SANS including: unilateral and bilateral optic disc edema, globe flattening, choroidal and retinal folds, hyperopic refractive error shifts, and focal areas of ischemic retina (i.e., cotton wool spots). We also discuss the knowledge gaps for in-flight and terrestrial human research including potential countermeasures for future study. We recommend that NASA and its research partners continue to study SANS in preparation for future longer duration manned space missions.

## Introduction

Unique and distinctive clinical and imaging findings occur in astronauts both during and after short and long duration space flight (LDSF). The term, “Space flight Associated Neuro-ocular syndrome” (SANS) has been used to describe these interesting findings. In the United States (US), the Space Medicine Operations Division of the National Aeronautics and Space Administration (NASA) has retrospectively and prospectively documented the findings of SANS.

Although a formal designation has not been validated, for the purposes of this review we consider the presence of any of the following findings post flight (compared to preflight) to be the working case definition of SANS: unilateral and bilateral optic disc edema (variable Frisén grades), globe flattening (as defined qualitatively on imaging), choroidal and retinal folds, hyperopic refractive error shifts (>0.75 D), or focal areas of ischemic retina (i.e., cotton wool spots). These findings in SANS have been documented over the past decade in multiple reports. In addition, structural changes have been documented that correlate with the clinical findings of SANS on ocular (e.g., optical coherence tomography [OCT]), orbital (e.g., ultrasonography and magnetic resonance imaging [MRI]) and cranial MRI. Visual acuity, Amsler grid, ophthalmoscopy, tonometry, fundus photography, orbital ultrasound and OCT are available on the International Space Station (ISS) and have been essential in documenting the development of the in-flight changes of SANS.^1–3^

We recognize and acknowledge the limitations of the current SANS definition and that the clinical syndrome has been re-defined over time as new information has emerged over the last decade. Mader et al. in 2011 originally described the clinical findings of SANS in astronauts after LDSF.^1^ In this initial cohort, seven astronauts received complete eye examinations before and after LDSF. Six astronauts received post-mission OCT and orbital/cranial MR imaging. Four of these astronauts underwent a lumbar puncture (LP) from 12 to 66 days following return to Earth. The only symptom noted was decreased near vision (hyperopic refractive shift) varying from +0.50 diopters (D) to +1.75 D (*n* = 6). No astronauts reported symptoms of increased intracranial pressure (ICP), including pulse synchronous tinnitus, double vision, or photopsias. The neuro-ophthalmic findings included optic disc edema of variable Frisén grade (*n* = 5), globe flattening (*n* = 5), choroidal folds (*n* = 5), cotton wool spots (*n* = 3) and retinal nerve fiber layer thickening on OCT (*n* = 6).^1^ Globe flattening (with secondary axial hyperopic shortening) was noted on terrestrial post-flight orbital MRI and orbital ultrasound (*n* = 5). An LP was performed in four individuals (total *n* = 6 LP: one astronaut had three LPs) and documented variable ICP measurements, ranging from borderline elevated to elevated opening pressures (OP). These included ICP measurements of 22 cm of water on day of return to Earth plus 66 days (*R* + 66), 21 cm of water (*R* + 19), 28 cm of water (*R* + 12), and 28.5 cm of water (*R* + 57).^1^ Although all affected astronauts were correctable to 20/20 visual acuity, some astronauts had persistent, residual refractive error changes for several years following LDSF.^4,5^ Persistent structural changes (e.g., choroidal folds, globe flattening) have also been seen on serial follow up MRI, ultrasound, OCT, and fundus examinations of astronauts from this initial cohort.^1,4,5^

### Potential etiologies and pathogenesis for SANS

Although the specific etiology of the optic disc edema, globe flattening, retinal and choroidal folds and hyperopic shifts described in SANS is unclear, two basic theories have been offered (although these are not mutually exclusive hypotheses). First, these changes may result from a rise in intracranial pressure (ICP) from cephalad fluid shifts during LDSF. In the terrestrial environment, CSF is largely produced in the choroid plexus and drains into the lower pressure cervical venous vasculature.^1–9^ Although vascular autoregulation stabilizes the cerebral and ONH arterial diameter,^10,11^ jugular venous distention has been well documented during both head down and microgravity (MG) studies^12^ suggesting that cerebral and jugular venous congestion may be present in the MG environment. Jugular venous distention alone does not necessarily prove that venous pressure is increased during spaceflight and some studies have suggested that the venous pressure may in fact be decreased. However, a spaceflight-induced decrease in CSF drainage into the venous system and cerebral venous congestion could potentially result in a rise in ICP that may be transferred down the orbital optic nerve sheaths resulting in optic nerve sheath expansion, stasis of axoplasmic flow and globe flattening similar to those changes that occur in patients with terrestrial idiopathic intracranial hypertension (IIH). Moderately elevated post LDSF lumbar puncture opening pressures of 28 and 28.5 cm of water measured post-flight in astronauts at 12 and 57 days respectively, which may have been higher during the mission, support this hypothesis.^1^ Also, MR imaging changes in astronauts following LDSF are suggestive of increased ICP, and include pituitary concavity, empty sella, and changes in pituitary stalk configuration.^13^ Several other factors have been hypothesized to cause or at least augment increased ICP during LDSF. These potential risk factors include high salt diets, rigorous resistive exercise, exposure to elevated ambient CO2 levels and possible defects in the vitamin B12-dependent 1-carbon transfer pathways.^14^

Several factors may speak against the ICP hypothesis as the sole explanation for the anatomical changes documented in astronauts. First, none of these astronauts with optic disc edema, globe flattening, choroidal folds, or hyperopic shifts presented with chronic severe headaches, transient visual obscurations or diplopia. Terrestrial IIH is typically associated with prominent headaches, seen in more than 90% of all IIH patients,^15^ but only mild or occasional headaches are reported in astronauts on ISS. Likewise, transient visual obscurations lasting seconds at a time are seen in 68% of IIH patients^15^ but have not been reported in astronauts. Diplopia from a non-localizing sixth nerve palsy has been documented in up to 30% of IIH patients^15^ but has never been reported in SANS. Second, most cases of terrestrial IIH present with bilateral and symmetric disc edema with highly asymmetric or unilateral disc edema documented in only 3–10% of IIH patients.^15,16^ In contrast, of five astronauts with ODE following LDSF in our 2011 report, one displayed highly asymmetric ODE, two had strictly unilateral ODE and only two displayed symmetric ODE. Third, if venous stasis from microgravity induced cephalad fluid shifts was solely responsible for astronaut disc edema we would expect this edema to quickly resolve following a return to the 1 G environment. However, we have documented persistent ODE in astronauts up to six months post mission.^5^ Fourth, although increased ICP may be a factor in SANS, the post LDSF values of available ICP measurements in astronauts have been just above normal to borderline high and do not seem to be in the markedly elevated range of ICP that we typically see in terrestrial IIH where ICP values can be as high as 30–40 cm of water or more. Finally, astronaut case reports also suggest that increased ICP alone may not be responsible for SANS. Specifically, asymmetric ODE in the setting of a normal ICP 1 week after space flight has been reported.^4^ In addition, unilateral loss of previously visible spontaneous venous pulsations during space flight has been described in an eye with ODE that continued to be absent for 21 months after return to Earth.^17^ Asymmetric disc swelling was described in another astronaut over 180 days post-flight and asymmetric optic disc morphologic changes persisted for 630 days post-flight in the presence of ICP measurements of 22 and 16 cm H20 obtained at 7 and 365 days post-flight respectively.^5^ Table 1 summarizes some of the key differences between terrestrial IIH and SANS. Renewed interest in ICP in SANS has led to a re-examination of both the on ISS (e.g., ultrasound) and on earth analogs for testing potential countermeasures for SANS.

**Table 1:** Features comparing and contrasting terrestrial idiopathic intracranial hypertension (IIH) and space flight associated neuro-ocular syndrome (SANS).

	IIH	SANS
ONH/Disc edema	Yes	Yes
Intracranial pressure (ICP)	Increased	Some elevated intracranial pressures on post-flight lumbar punctures but inconclusive evidence that increased ICP is the major etiology
Female:Male ratio	9:1	No females officially diagnosed (0:10), but ocular changes detected in both sexes
Body habitus	Obese (>90%)	Normal to highly athletic
Symptoms	Chronic headaches (94%); Transient vision obscuration (68%); Pulse synchronous tinnitus	Typically none besides vision complaints (Near > Distance)
Side bias	<4% unilateral	To be determined; but gross signs have been right-biased
Radiographic findings	Gross anterior movement of fluid within the subarachnoid space, optic nerve sheath, flattening of the globe, empty sella, venous sinus stenosis without thrombosis	Increased fluid within the orbital subarachnoid space and sheath, flattening of the globe, cephalad brain shift, limited evidence for venous sinus abnormalities
Retinal: Bruch’s membrane and choroidal and retinal folds	Bruch’s membrane opening anterior movement. 5:1; Retinal folds occur first	Bruch’s membrane opening posterior movement and 1:5; Choroidal folds occur first
Fold pattern	Typically concentric around ONH (Paton’s lines)	Typically linear

The optic disc edema seen both in terrestrial models of increased ICP and in SANS demonstrates a pattern of nerve fiber layer thickening consistent with papilledema. Invasive monitoring in humans during spaceflight have not been performed but such changes were transient and mild in a macaque monkey.^18^ Terrestrial idiopathic intracranial hypertension (IIH) is an imperfect analogy for SANS (Table 1). The prelaminar NFL edema (seen in terrestrial papilledema) presents as positive anterior angulation of the Bruch membrane opening but in SANS shows negative, posterior retroorbital angulation. Although astronauts demonstrate indirect radiographic evidence of increased ICP with optic nerve sheath distension this does not adjudicate between the competing hypothesis of cephalad and orbital fluid shifts. In addition, sustained papilledema from increased ICP typically leads to optic atrophy following the resolution of the axonal swelling which has not been demonstrated in SANS. Sustained ICP elevation is correlated with increased levels of HIF-1α in the retinal ganglion cell layer, and some models have demonstrated presumed breakdown of the blood retinal and blood optic nerve barrier with vascular leakage into the interstitial space of the ONH and subretinal space in the peripapillary region.^19^ In addition, increased ICP that resolves upon return to the terrestrial 1 G environment might produce other local structural changes that might induce secondary inflammation or oxidative stress and the residual optic nerve sheath distension and choroidal folds after return might suggest that the elasticity of the optic nerve sheath trabecular fibers or collagen structures might be permenantly altered by spaceflight independent of ICP. Further studies are needed to determine the role if any of local vascular stasis, inflammation, or oxidative stress to the pathophysiology of SANS in relation to ICP.

A second possible explanation for SANS is compartmentalization of CSF within the orbital optic nerve sheath.^1,5^ In the past, it was generally assumed that there was homogeneity of ICP and chemical components of the CSF throughout the brain, spinal cord and orbital optic nerve sheath. However, the unique tightly confined and cul-de-sac like anatomic connection between brain and orbit in the optic nerve sheath may create a fragile flow equilibrium and a possible one-way valve like effect that may lead to pressure elevation within the orbital optic nerve sheath with or without elevated CSF pressures surrounding the brain.^1,4,5,20–23^ It has also been proposed that a microgravity induced glymphatic flow imbalance within the orbit may play a role in this process.^24–26^ The compartmentalization theory has been previously proposed to explain the existence of continued disc edema in terrestrial patients with functional lumboperitoneal shunts.^1,21,22^ Shinojima recently offered an alternate compartmentalization theory which proposes that during LDSF the optic nerve and globe may be retracted posteriorly as a result of brain upward shift and resultant uplifting of the optic chiasm during and after LDSF.^27^ They propose that this posterior “pull” on the optic nerve and globe compresses the CSF within the optic nerves leading to localized pressure elevation and expansion.^27^ This spectrum of potential mechanisms emphasizes the possible multifaceted origin of the unusual neuro-ophthalmic findings in SANS.^1,5,20–22,27^

Initially, due to the moderately elevated ICP measurements in some astronauts, SANS was termed the visual impairment and intracranial pressure (VIIP) syndrome. However, over time the role of elevated ICP as the sole mechanism for the findings has come into question and the name was changed from VIIP to SANS to reflect the uncertainty about the pathogenesis and the possible multifactorial etiology for the findings. Although the complete pathologic process may be multifaceted and somewhat variable from astronaut to astronaut, we believe that locally elevated unilateral or bilateral CSF sheath pressure within the orbit, resulting from cephalad fluid shift related phenomena, is likely an important mechanism for the ocular and imaging findings of SANS as opposed to elevated ICP alone. Unfortunately, pre-flight LP data are not available in any astronauts including prior ISS flyers. Ongoing discussions at NASA have centered on the possibility however of obtaining pre-flight ICP measurements.

Some head down and MG studies have documented that cerebral arterial diameter and blood flow velocity are autoregulated and do not change significantly during space flight.^10,11^ It is not known if the middle cerebral artery diameter changes significantly during spaceflight, and other studies have concluded that cerebral autoregulation may be impaired during spaceflight.^28^ However, microgravity fluid shifts during spaceflight (including cephalad fluid shift to the head and orbit) have been documented to cause jugular vein distension, as well as mild OCT thickening of the retinal nerve fiber layer of the optic nerve.^26,29–31^ Interestingly, these same OCT changes have been demonstrated in head down tilt (HDT), bed rest studies on earth,^32^ and HDT studies are believed to be a reasonable terrestrial analog for the cephalad fluid shift in microgravity in SANS. The mechanics of ICP and the possible role of glymphatics and the venous system in SANS however remains ill-defined but have sparked additional debate and hypotheses.^24,25,33^

Terrestrial and in-flight ISS OCT have demonstrated more widespread ocular changes than seen on clinical exams alone. As of December 2018, additional OCT capability is available on ISS, and the use of enhanced depth imaging (EDI) OCT, OCT2, MultiColor Imaging, and OCT angiography (OCTA) will increase our sensitivity for detecting the spectrum of structural changes seen to date with conventional spectral domain OCT in SANS. OCT2 is the next generation of OCT and offers enhance resolution and depth imaging from vitreous to choroid. OCT2 also has additional features of improved patient reliability and is more operator friendly. The cephalad fluid shift theory proposes that venous congestion in the orbit, neck, and head might lead to elevated vortex vein pressures, decreased choroidal drainage and stagnation or pooling of blood in the choroid, and secondary choroidal expansion, elevated IOP, globe flattening, and choroidal folds. Documenting these potential choroidal volume changes in a more detailed and quantitative manner with newer OCT technology will be important going forward.^13,25,34–37^

### Orbital and cranial MRI and orbital ultrasound findings in SANS

In the years following the discovery of SANS, astronauts began receiving high-resolution, 3-Tesla (3 T) magnetic strength MR imaging of the head and orbits, prior to and as soon as possible after spaceflight. MR imaging is obtained 18–21 months prior to LDSF and within three days after the crewmember’s return. Two-dimensional ocular ultrasound is obtained 6–9 months prior to LDSF, on-orbit (typically at flight days 30 and 90, and 30 days prior to return), and within 3 days post-flight. There are data which overlap between MR and ultrasound imaging modalities; however each has its advantages, and of the two, only ocular ultrasound is available onboard the ISS. Table 2 provides a general overview of the detectability of SANS signs by the primary devices and tests utilized by NASA for SANS diagnostics. We describe the qualitative and comparative sensitivity and specificity of the various available tests for detecting specific signs in SANS and not the positive predictive value for any one modality for predicting SANS. Because there is no gold standard for some of the SANS findings (e.g., MRI demonstrated globe flattening) we can only provide our subjective assessments of the utility of the NASA available tests for SANS.

**Table 2:** Detectability of SANS signs by diagnostic device/test.

	Diagnostic device/test				
SANS sign	OCT	Fundus	MRI	US	VF
Optic disc edema	+++	++	Very low sensitivity	–	Indirect indication (if enlarged blind spot or scotoma detected)
Retinal nerve fiber thickening	+++	–	–	–	–
Chorioretinal folds and peripapillary wrinkles	+++	Low-to- moderate sensitivity	–	–	–
Cotton wool spots	High sensitivity (with MultiColor Imaging)	High sensitivity and specificity	–	–	–
Retinal hemorrhages	NA		–	–	–
Globe flattening	–	–	High sensitivity	Low-to- moderate sensitivity	Indirect indication
Refractive error shift	–	–	Indirect indication	High sensitivity	–

*OCT* optical coherence tomography, *MRI* magnetic resonance imaging, *US* ultrasound, *VF* visual field, *NA* not applicable.

One clinical sign of SANS is globe flattening, where the convexity of the posterior sclera is reduced compared with the spherical shape of the remainder of the normal globe. Posterior globe flattening may persist for years post-flight. Globe flattening also decreases the axial length of the globe and drives an anterior displacement of the fovea, which induces a hyperopic shift in refractive error. Globe flattening has been documented by comparison of pre and post LDSF axial length measurements as well as hyperopic shifts in refraction. In addition, qualitative globe flattening is well documented by MR imaging and ocular/orbital ultrasound. Orbital ultrasound is currently used on the ISS for this purpose but at a lower resolution and sensitivity than high field strength orbital MRI. We recognize however that the reading of both MRI and orbital ultrasound is somewhat subjective and that our interpreting clinicians are not masked as to prior flight status. Countermeasures to reduce over-reading bias, including single or multiple and masked readings, have not been performed to date by NASA but could be considered in the future. We recognize the inherent bias in unmasked MRI readings for posterior globe flattening even by experienced neuroradiologists. Unfortunately, the readers are not masked to flight status and no formal bias mitigation strategies (e.g., multiple readers, single, or double masking, etc.) are employed by NASA when classifying an astronaut as a SANS case. Perhaps this could be considered in the future however.

While OCT technology is vastly superior in detecting anatomical changes within the optic nerve (ON) head, optic nerve protrusion can also be visualized by both MR and ultrasound imaging. Indeed, after analyzing post-flight MR images in 15 LDSF and 12 short-duration spaceflight (SDSF) astronauts, Kramer et al.^13^ confirmed the presence of post-flight optic nerve protrusion, with all four cases being associated with LDSF and none with SDSF. In addition, these researchers analyzed optic nerve sheath diameter (ONSD), and suggested that the average ONSD of veterans (mean = 6.2 ± 1.1 mm) is near or greater than what is predictive of intracranial hypertension (>25 mm H_2_O) in terrestrial patients (i.e., a 5.0–6.0 mm cutoff). ONSD was greatest for those astronauts presenting with posterior globe flattening (mean ONSD = 7.2 ± 1.5 mm; *n* = 7) or optic nerve kinking (mean ONSD = 7.5 ± 1.1 mm; *n* = 4). Sirek et al. also have used Doppler ultrasound to document ONS expansion during HDT and during spaceflight.^38^ It should be noted that while intraorbital optic nerve tortuosity and kinking are potential anatomical signs of SANS, only a limited number of crew members currently possess baseline MR images that permit thorough evaluations the optic nerve (private communication William Tarver, MD). Therefore, confirmation of any pre-to-post-flight changes in optic nerve tortuosity or kinking, or any conclusive associations between these radiographic signs and SANS have yet to be established. Orbital ultrasound however can be used to demonstrate optic nerve tortuosity and there are ongoing efforts to document these findings on ISS compared to pre-flight baseline.

Few published studies have investigated intracranial findings associated with spaceflight. Kramer et al.^13^ noted that, in post-flight MR images, 3-of-15 of LDSF and 0-of-12 SDSF astronauts exhibited moderate or greater pituitary dome concavity with posterior stalk deviation. The authors noted that in terrestrial patient populations, these signs can be associated with altered CSF dynamics and intracranial hypertension. Roberts et al.^39^ studied the pre-flight and post-flight MR brain images for 18 LDSF and 16 SDSF astronauts and discovered a narrowing of the central sulcus in 17 and 3 of the two cohorts, respectively. In a subgroup that underwent additional three dimensional (3-D) T_1_-averaged MR imaging, 12-of-12 LDSF astronauts experienced an upward shifting of the brain and a narrowing of the vertex CSF spaces, while 0-of-6 SDSF astronauts expressed an upward brain shift, and only 1-of-6 showed a narrowing of the vertex CSF spaces. The percentage change in the total volume of the ventricular system (lateral, third, and fourth ventricles) between pre-flight and post-flight was quantified in the 18 participants for whom high resolution, three-dimensional, T1-weighted sequences were obtained. This subgroup was also analyzed for pre-flight to post-flight percent changes in ventricular system total volume, and a significant difference was found between the LDSF (+11 ± 5.9%) and SDSF (+0.04 ± 1.87%) groups.

Koppelmans et al.^40^ detected significant decreases in gray matter (GM) volume from pre-flight to post-flight in a group of 27 astronauts. These decreases were located around the frontal and temporal poles, and near the orbits. They also observed small, localized GM increases in the medial primary somatosensory and motor cortex. Overall, the authors suggest that these changes in GM volume may be related to cephalad fluid shifts or neuroplasticity. Van Ombergen et al.^41^ summarized a prospective MR imaging study performed on ten male LDSF cosmonauts, where images were obtained pre-flight, promptly upon return (mean = 9 days post-flight), and multiple months after return (mean = 209 days post-flight; *n* = 7). Like Koppelmans et al.^40^ the authors described changes in pre-flight to post-flight GM volume, with a widespread decrease in the orbitofrontal and temporal cortexes. The greatest decrease (3.3%) occurred in the right middle temporal gyrus. Long-term images suggested a recovery of GM volume towards pre-flight levels, although a GM volume reduction (1.2%) persisted in the right temporal gyrus. Similar to Roberts et al.^39^ short-term post-flight CSF volume was reduced below the vertex, and ventricle volume was found to be increased (with a maximum increase in the third ventricle [12.9%]). By the long-term follow-up date, ventricle volumes had almost completely recovered toward pre-flight values, while CSF volume increased within the entire subarachnoid space surrounding the brain. The authors suggest that these findings might relate to clinical findings associated with long-duration spaceflight.

With a steadily increasing number of high-resolution pre-flight and post-flight MRI becoming available for analysis, additional in-depth investigations will be possible. At the present time, it is not known if these intracranial findings are associated with any functional/performance changes, or if they even fall within the SANS spectrum. Future efforts may include clinical, OCT, and MRI examinations of space naïve astronauts (i.e., crew members who have not flown previously in space) in order to avoid the possible impact of previous MG exposure on ocular and cerebral anatomy. Results of pre-flight testing in these astronauts could be compared with both normal controls and with terrestrial patients with IIH.

Given the abundance of post-flight clinical findings in NASA crew members, NASA has been considering the incorporation of more advanced MR sequences into the standard pre-flight and post-flight astronaut imaging protocols to discover the elements responsible for micro-gravity induced brain changes in the astronauts. Currently, only basic volumetric T1-weighted and T2-weighted MRI images are performed. Visual impairment has been recognized as one of the most impactful astronaut health risks which needs to be addressed as we plan for very long-term spaceflights such as travel to the moon or Mars.

Undoubtedly, comparison of the pre-flight and post-flight high-resolution anatomical MR images of the orbits and the brains of astronauts has significantly helped us to better assess and understand the post-flight changes. It is worth noting that in addition to all these efforts, some advanced MRI techniques such as Diffusion Tensor Imaging (DTI) have been recently utilized to identify the microstructural change in SANS in the posterior part of the visual system which may contribute to the bigger picture of “post-flight visual impairment” in the crewmembers.

A recent quantitative comparison of pre-flight and post-flight brain MR scans of 19 astronauts was performed implementing the anatomical and DTI sequences.^42^ Basically, diffusion-tensor MR imaging and fiber tractography are MRI techniques to estimate the axonal organization of the brain. Diffusion tensor metrics provides a quantitative analysis of the magnitude and directionality of water molecules. In this regard, mean diffusivity (MD) reflects the average magnitude of molecular displacement by diffusion where the greater values are indicative of a more isotropic medium. Fractional anisotropy (FA) also shows the directionality of this diffusion and varies between 0 (isotropic diffusion) and 1 (infinite anisotropic diffusion). Any change in these parameters is mainly related to axonal integrity, myelination, axon diameter and density. Evaluation of the white matter tracts in the above study demonstrated a decrease in the FA in the right posterior thalamic radiation in the astronauts’ brain after their space-missions (*p* = 0.0009, *p* = 0.03 after FDR correction). A trend of increase in the MD in the gray matter of the right calcarine, middle occipital, inferior occipital and fusiform gyri was also recorded by this group in the post-flight scans. The identified changes in this study were primarily attributed to cerebral edema and fluid redistribution in the visual pathways secondary to the effect of intracranial hypertension induced by microgravity. Furthermore, brain morphometric analysis of post-flight scans showed a trend of decrease in the mean of cortical thicknesses in the right occipital and bilateral fusiform gyri.

Another retrospective analysis of the brain MRI scans of 15 astronauts has also shown significant widespread spaceflight-associated free water increases at the base of the cerebellum and decrease along the posterior vertex which was reflecting disrupted white matter structural connectivity in the cerebellum, corticospinal tract and superior longitudinal fasciculus, among other regions after adjustment.^43^ Unfortunately, some of the clinical and radiographic findings in SANS may not be unique to only LDSF because many of the astronauts thus far studied had previous SDSF and thus had no true pre-flight comparative data. In addition, some of the MRI findings (e.g., globe flattening) may be subtle and could easily be over-interpreted especially if the neuroradiologist is not masked to their flight status.

In order to effectively link these brain microstructural changes in SANS to clinical findings, future masked imaging studies will be needed. Longitudinal analysis of the brain MR scans of the astronauts in different time-points should also help to elucidate recovery time course and reversibility of described changes.

### OCT and SANS

Since its deployment to the ISS in 2013, NASA researchers have been using the Heidelberg Spectralis OCT imaging platform to study the effects of microgravity on posterior ocular structures. OCT is a noninvasive imaging test that uses light waves to take a high resolution cross-section picture of the retina, choroid, and optic nerve head (ONH), and has become the primary diagnostic tool for the early detection and monitoring of SANS. The standard for assessing clinical optic disc edema has been the Frisén grading scale system as quantified by a dilated eye exam or fundus photography. High resolution OCT technology has had a significant impact on quantifying early morphological changes of the posterior ocular structures and detecting ocular pathology (e.g., IIH, glaucoma, macular degeneration).

**Fig. 1:**
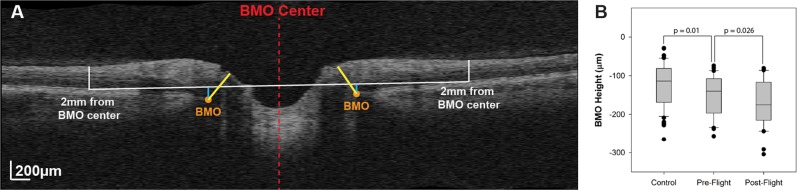
Bruch membrane opening (BMO) and height. **a** Marking of the BMO (orange marker) is shown on 1 radial section through the optic nerve head. The BMO center (red dashed line) was used to determine the location for a reference plane at 2 mm (white line), from which the BMO height was quantified (blue line). **b** The BMO height is recessed in pre-flight optical coherence tomographic (OCT) scans compared with healthy controls. This difference increases after long-duration microgravity exposure. It should be noted that most astronauts included in this study had previous spaceflight experience. (Reprinted with permission from Patel et al.^87^).

**Fig. 2:**
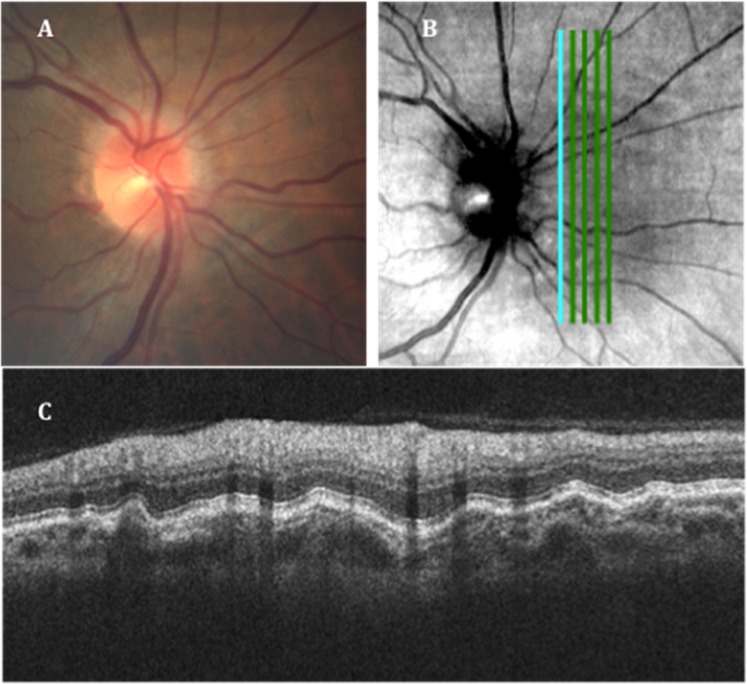
Post-flight imaging of the right eye. **a** Fundus imaging showing the “C” halo of the Frisén Grade 1 disc edema and choroidal folds inferior to the disc. **b** Scanning laser ophthalmoscopy (SLO) image from the SD-OCT with an overlay of the vertical raster scan placement. Note the choroidal folds superior and inferior to the disc visible in the SLO image. **c** The retinal cross-section obtained by the vertical scan just nasal to the disc showing retinal nerve fiber layer thickening and severe choroidal folds. (Reprinted with permission from Patel et al.^87^).

Patel et al. published a retrospective study comparing OCT (Cirrus HD, Carl Zeiss Meditec) analysis of the ONH and surrounding structures in 15 long-duration ISS astronauts with 43 healthy control subjects with no history of ocular pathology or microgravity exposure.^44^ Patel et al. showed an increase in retinal nerve fiber layer (RNFL) thickness and total retinal thickness close to the optic nerve head margin consistent with optic disc edema, and a posterior shift in Bruch’s membrane opening (BMO) position as quantified by BMO height in astronauts after microgravity exposure (Fig. 1).^44^ The study also revealed a significantly high proportion of eyes with choroidal folds in astronauts after LDSF (Fig. 2), while no choroidal folds were detected on healthy controls. The changes in retinal nerve fiber layer thickness and total retinal thickness are similar to those observed with terrestrial IIH. However, the posterior deflection of the BMO height and the relatively high incidence of choroidal folds are not commonly seen with IIH, suggesting an alternate hypothesis for SANS. As the location of the BM may be influenced by the underlying choroid, perhaps changes in choroidal volume during LDSF may impact the BMO angle in astronauts and be part of the multifaceted pathogenesis for SANS. The results of this study also suggest that although there may be some resolution of structural changes, there can be long-term anatomical changes after extended-duration spaceflight.

To date approximately 15% of astronauts had clinically significant (Frisén grade 1–3) ODE after LDSF (private communication William Tarver, MD). However, we would like to emphasize that OCT has revealed some level of ODE in nearly all astronauts when comparing pre-flight and in-flight OCT and this suggests that subclinical SANS involvement may occur in the majority of astronauts. Choroidal folds have been observed in over 20% of astronauts following LDSF and in-flight OCT has documented choroidal thickening on all tested astronauts compared with pre-flight baseline OCT.^5^ Ongoing studies that include OCT imaging of the posterior ocular segment on the ISS will provide additional information on the etiology and time course of these structural changes that will be important not only for understanding the pathophysiology of SANS but also for developing countermeasures. In addition, OCT algorithms and methods used to determine ocular morphological changes in astronauts could be helpful in both future space travel studies as well as related clinical studies here on Earth.

### NASA current and future operations and research for SANS

NASA astronauts undergo extensive medical evaluations at the time of selection and routinely thereafter. Although medical (including ophthalmic) testing related to SANS has been ongoing over the last decade, little SANS-specific data were acquired prior to 2010. Additionally, early pre-flight data were potentially skewed due to the vast majority of astronauts having had already flown at least one SDSF or LDSF. For example, all seven of the astronauts reported in the 2011 Mader paper^1^ had previous spaceflight experience, and none had the SANS-specific MR imaging performed prior to their last flight. Thus, clinical imaging and anatomical changes due solely to LDSF could not be accurately determined.

More recently, however, pre-flight data have been collected on space naïve astronauts. As this unbiased dataset becomes available for analysis, the true nature of the changes seen due to LDSF alone can be evaluated. Two case reports^2,3^ exemplify how such data can be useful. Astronaut medical and research data are archived in the Lifetime Surveillance of Astronaut Health (LSAH) repository and the Life Sciences Data Archive (LSDA) repository, respectively. Information about these archives is available at a website portal (https://lsda.jsc.nasa.gov/).

Limitations related to space flight impede medical and research data in several ways. The current astronaut return to Earth is a hard landing in the former Union of Soviet Socialist Republics (USSR) country of Kazakhstan. This creates challenging political, operational, and logistical barriers to performing a timely, accurate and safe LP on returning astronauts. Recently, more direct air transportation back to Houston, Texas, USA from Kazakhstan gives a better window of opportunity for performing post-flight LPs and measuring true ICP values closer in time to the actual return.

At present, three astronauts/cosmonauts launch about every 3 months, resulting in only 12 individuals flying annually. This limits study sample sizes. Although astronaut training backgrounds are rigorous and selected participants include aviators, scientists and physicians, none are experienced eye specialists. Astronauts receive about 7.5 h of SANS-related training in the year prior to their launch. During a 6-month mission, an astronaut will perform only a few SANS-related data collections, and each one is 30–90 days apart. Due to operational requirements and other competing tasks, medical operations testing and data collections must be kept simple and focused. Finally, for Orion missions beyond low earth orbit (LEO), the Space Medicine Operations Division of NASA is currently facing a weight constraint of 30 pounds for all medical equipment and supplies, and therefore, must judiciously choose the number, frequency, and extent of future medical exams.

Ongoing analysis has yet to clearly define the pathophysiology leading to SANS associated findings, and the current data do not point to a single pathologic mechanism (e.g., elevated ICP or cephalad fluid shift alone). Measuring changes in ICP before, during and immediately post-flight has never been performed in astronauts. In addition, unfortunately no valid and reliable measures (either direct or indirect) of in-flight ICP have been available for use during spaceflight. Post-flight ICP data have been acquired only in astronauts who experienced optic disc edema clinically. Thus, no astronaut population-based ICP data exist although NASA is working diligently to determine a forward path toward solving this significant knowledge gap.

OCT figures prominently in our SANS surveillance scheme. OCT has a particularly prominent role at present as a high-quality objective measure of the optic nerve head (optic nerve head), macula and choroid complex. Unlike ICP, OCT data can be gathered in a rapid, noninvasive, safe, reliable, and reproducible manner during pre-, in- and post-flight testing.

**Fig. 3:**
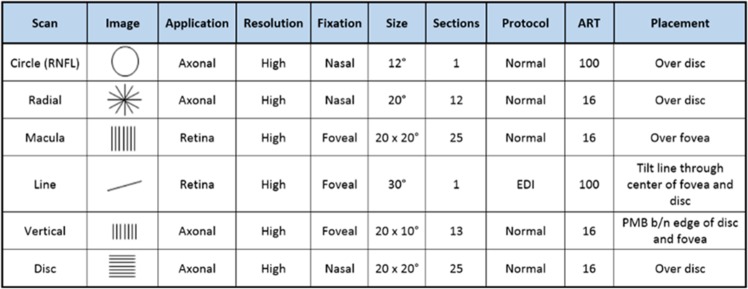
Prior optical coherence tomography (OCT) scanning protocol for SANS.

**Fig. 4:**
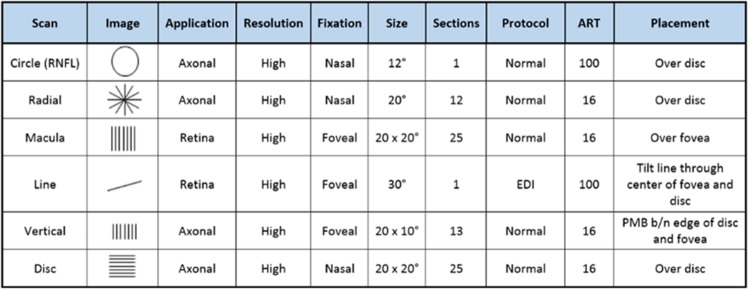
Current OCT2 scanning protocols for SANS.

A Heidelberg Spectralis “OCT2” device has been recently activated on ISS and promises acquisition of denser imaging of the optic nerve head, retina, and choroid. Figure 3 demonstrates the current SANS OCT protocol (2014–2019) compared to the proposed SANS OCT protocol in Fig. 4.

The upgraded OCT2 on the ISS has another feature that will replace routine screening fundoscopy during missions—multicolor imaging (MCI). This false color image technique may provide more useful data and can be supplemented with fundoscopy when clinically indicated. A yet-to-be-published ground-based study conducted by the Doheny Eye Institute in 2018 compared fundoscopy to MCI, and results indicate that SANS signs that are visible by fundoscopy are equally detectable by MCI (personal communication Tyson J. Brunstetter OD).

The breathing environment on ISS is 14.7 psi with normal oxygen and nitrogen levels. However, technical constraints maintain the carbon dioxide (CO_2_) levels at nearly 10 times Earth normal. Since astronauts are exposed to these elevated CO_2_ levels in addition to MG the possible physiologic role of CO_2_ remains ill defined. Elevated radiation levels are also of concern on ISS and the potential role for radiation during LDSF and its potential role in SANS likewise remains an area of great interest. It should be noted that CWS have been documented in several astronauts following LDSF.^1,5^ As these are also seen following terrestrial radiation therapy^45^ their presentation in astronauts following LDSF suggests^46–54^ the possible role of space radiation exposure.^5^ NASA is attempting to tease apart the role that each of these elements may play in SANS. Law et al. have compared headaches against measured CO_2_ levels.^55^ Also, genetic variations in the one-carbon metabolism pathway and the potential role for these genetic predispositions and their significance are being explored by several research groups.^14^

Terrestrial and in-flight ISS OCT have demonstrated more widespread ocular changes than seen on clinical exams alone. We and many other authors believe that choroidal expansion from microgravity-related cephalad fluid shifts might be an additional mechanism to explain the globe flattening, hyperopic refractive shift, and retinal and choroidal folds seen on OCT. OCTA, which was not available previously on ISS, has demonstrated promising potential for retinal and choroidal disease on earth and may prove useful in defining retinal and choroidal vessel changes in SANS. The role of OCTA in the future evaluation of SANS, however, remains to be defined. The cephalad fluid shift theory proposes that venous congestion in the orbit, neck, and head during spaceflight might lead to elevated vortex vein pressures,^1,35–37,39,56,57^ decreased choroidal drainage and stagnation or pooling of blood in the choroid, with secondary choroidal expansion as well as globe flattening, and set the stage for choroidal folds. Documenting these potential choroidal volume changes in a more detailed and quantitative manner with newer OCT technology will be important going forward.

### Revisiting ICP and potential countermeasures for the future

As noted above, the neuro-ocular findings of SANS were initially termed the visual impairment, intracranial pressure (VIIP) syndrome. In response to a gradual reduction in the presumed role of ICP in the pathogenesis, SANS was chosen as a more appropriate descriptive term. Recently however the possible role of ICP and translaminar pressure differences between ICP and intraocular pressure (IOP) have been of more interest.^18,58,59^ Lawley et al. documented that prolonged periods of simulated microgravity did not cause progressive elevations in ICP. He suggested that complete removal of gravity does not pathologically elevate ICP but does prevent the normal lowering of ICP when upright. His findings suggested that the terrestrial human brain is protected by the daily circadian cycles in regional ICPs, without which pathology may occur. He speculated that the absence of diurnal, postural reductions in ICP relative to IOP in microgravity creates a persistently lower pressure gradient at the posterior aspect of the eye that may result in optic remodeling.^60^ The ongoing debate has prompted revisiting some of the other risk factors including carbon dioxide and in particular the role of hypercapnia in increased ICP and in terrestrial analogs (e.g., head down tilt studies) that may mimic the cephalad fluid shift. Renewed interest in the role of ICP^60–69^ in SANS has led to discussion about the feasibility of performing a lumbar puncture in space despite the obvious logistical and operational challenges to an in-flight invasive procedure.^70–72^ Non-invasive assessment of ICP have not been reliably validated on earth but ultrasound is available on the ISS and the possibility of ultrasonographic metrics (e.g., optic nerve sheath diameter, CSF in the sheath) remain an area of continued research. Likewise terrestrial head down studies with and without hypercapnia have demonstrated increased retinal nerve fiber layer on OCT and might still prove to be a suitable terrestrial analog to test hypotheses and potential countermeasures to SANS.^73–82^ These countermeasures include metabolic and pharmacologic treatments^69,83–87^ in the one carbon pathways;^63,69^ lower body negative pressure;^74–76^ and swim goggles to affect the translaminar pressure gradient. While current data does not seem to support prolonged, significant elevations of ICP to the levels seen in IIH, throughout LDSF, Lawley et al. proposed that even mild elevations of ICP for a prolonged duration may contribute to the structural changes seen in SANS. Table 3 outlines some of the proposed areas for continued research in SANS.^60,78^

**Table 3:** Potential mechanisms of spaceflight associated neuro-ocular syndrome (SANS).

1. Cephalad fluid shift with intraorbital and intracranial volume increase
2. Increased intracranial pressure
3. Translaminar pressure gradient
4. Altered glymphatic drainage
5. Intracerebral volume and cerebral edema alterations
6. Orbital and cerebral arterial or venous drainage disturbance
7. One carbon pathway metabolism alterations
8. Choroidal volume expansion
9. Hypercapnia related volume and pressure disturbances

## Conclusion

In summary, novel and unique neuro-ophthalmic findings have been documented in astronauts during and after LDSF and have been termed SANS. Although a single unifying and overreaching mechanism has yet to be proven,^1,2,4–9,19,40,41,44,55–57^ and a multifactorial pathogenesis may be involved, it is likely that SANS may be the end result of cephalad fluid shifts to the brain and orbit brought about by extended MG exposure. Mao et al. reviewed the impact of spaceflight and artificial gravity in a mouse retinal model using biochemical and proteomic analysis. Zhang and Hargens reviewed the possible role of spaceflight-induced intracranial hypertension and visual impairment as well as potential pathophysiology and Countermeasures. Wostyn and De Deyn described intracranial pressure-induced optic nerve sheath response as a possible predictive biomarker for optic disc edema in astronauts. The interested reader is directed to both our own prior review articles but also the animal and human work performed by the many intramural and extramural NASA related partners working on SANS.^6–9^

Despite the recognition and research related to SANS for many years, several unanswered questions remain: (1) What, if any, is the significance of potential preferential laterality (i.e., right-sided bias) seen in the anatomical changes of SANS? (unpublished data, personal communication WT); (2) Are there changes in the eye’s anterior segment (in addition to the posterior segment findings) associated with SANS?; (3) What, if any, is the role of CO_2_ or radiation in SANS?; (4) Are there pre-flight anatomic, hormonal or physiologic characteristics of in astronauts that predispose to the development or severity of SANS?; (5) Are there more sensitive and objective measures of the ODE in SANS (as compared to the Frisén scale)?; (6) Is there a dose (i.e., duration of LDSF) response curve or dose gradient for exposure with longer missions producing a greater SANS risk?; and (7) What role, if any, does the lymphatic system play in SANS? These and many other questions remain under active investigation by NASA and its clinical and research partners.

Understanding the possible mechanisms for SANS will undoubtedly be useful in developing preventive or counter measures before or during LDSF especially as NASA prepares for the possibility of even longer duration manned missions to the ISS, the moon, the asteroid belt, or Mars.
