# Author Correction: Spaceflight associated neuro-ocular syndrome (SANS) and the neuro-ophthalmologic effects of microgravity: a review and an update

**DOI:** 10.1038/s41526-020-00114-8

**Published:** 2020-08-26

**Authors:** Andrew G. Lee, Thomas H. Mader, C. Robert Gibson, William Tarver, Pejman Rabiei, Roy F. Riascos, Laura A. Galdamez, Tyson Brunstetter

**Affiliations:** 1 Department of Ophthalmology, Houston Methodist Hospital, Houston, TX USA; 2 Baylor College of Medicine and The Baylor Center for Space Medicine, Houston, TX USA; 3 Departments of Ophthalmology, Neurology, and Neurosurgery, Weill Cornell Medical College, New York, NY USA; 4 Department of Ophthalmology, The University of Texas Medical Branch, Galveston, TX USA; 5 Department of Ophthalmology, The University of Iowa Hospitals and Clinics, Iowa City, IA USA; 6 UT MD Anderson Cancer Center, Houston, TX USA; 7 Colonel (R) US Army, Moab, UT USA; 8 Coastal Eye Associates, Webster, TX USA; 9 Space Flight Associated Neuro-ocular Syndrome (SANS) Clinical Lead, Clinical Services, NASA Johnson Space Center, Houston, TX USA; 10 Department of Diagnostic and Interventional Imaging, University of Texas Health Science Center at Houston, Houston, TX USA; 11 U.S. Navy Detailed to NASA Johnson Space Center, Houston, TX USA; 12 Department of Emergency Medicine, Baylor College of Medicine, Houston, TX USA

**Keywords:** Eye manifestations, Medical research, Physical examination

Correction to: *npj Microgravity* 10.1038/s41526-020-0097-9, published online 7 February 2020

In the original version of this Article, the wrong version of Figure 4 was published. This has now been corrected in both the HTML and PDF versions of the Article.

